# Nanoscaled eutectic NiAl-(Cr,Mo) composites with exceptional mechanical properties processed by electron beam melting

**DOI:** 10.1038/s41598-020-72093-5

**Published:** 2020-09-16

**Authors:** Andreas Förner, S. Giese, C. Arnold, P. Felfer, C. Körner, S. Neumeier, M. Göken

**Affiliations:** 1 Department of Materials Science and Engineering, Institute I, General Materials Properties, Friedrich-Alexander-Universität Erlangen-Nürnberg (FAU), Martensstraße 5, 91058 Erlangen, Germany; 2 Chair of Materials Science and Engineering for Metals, Department of Materials Science and Engineering, Friedrich-Alexander-Universität Erlangen-Nürnberg (FAU), Erlangen, Germany

**Keywords:** Structural properties, Nanoscale materials, Composites, Structural materials, Mechanical properties

## Abstract

Eutectic NiAl-(Cr,Mo) composites are promising high temperature materials due to their high melting point, excellent oxidation behavior and low density. To enhance the strength, hardness and fracture toughness, high cooling rates are beneficial to obtain a fine cellular-lamellar microstructure. This can be provided by the additive process of selective electron beam melting. The very high temperature gradient achieved in this process leads to the formation of the finest microstructure that has ever been reported for NiAl-(Cr,Mo) in-situ composites. A very high hardness and fracture toughening mechanisms were observed. This represents a feasibility study towards additive manufacturing of eutectic NiAl-(Cr,Mo) in-situ composites by selective electron beam melting.

## Introduction

As early as the 1970s eutectic NiAl-Cr in-situ composites have been investigated by Walter and Cline^1–3^ and other researchers as potential candidates for advanced intermetallic materials^4,5^. The intermetallic Nickel-Aluminum (NiAl) B2-phase exhibits a low density of 5.9 g/cm^3^, excellent oxidation resistance up to 1,300 °C and good thermal conductivity of 92 W/mK, beneficial for high temperature applications^6,7^. However, the fracture toughness and creep strength are rather poor. The addition of 34 at.-% Chromium results in a eutectic alloy containing a two-phase microstructure. The so-called in-situ composite is able to increase the room temperature fracture toughness from around 3 MPam^1/2^ for pure NiAl up to 20 MPam^1/2^^8–10^. Furthermore, the high eutectic melting point of 1,445 °C is beneficial for high temperature structural materials in order to compete with the established Ni-base superalloys^11^. The mechanical properties strongly depend on the eutectic microstructure, where rod and lamellar microstructures have been reported. NiAl-34Cr consists of Cr rods embedded inside a NiAl-matrix^3^. There is a cube on cube orientation relationship between the B2 NiAl and the bcc α-Cr-phase with only a small lattice mismatch. Small amounts of Molybdenum (Mo) can be added to the alloy to substitute Cr atoms and increase the lattice parameter. That cancels out the lattice misfit at 0.6 at.-% Mo and inverts it for further addition. These semi coherent interfaces promote the microstructural modification towards alternating NiAl- and (Cr,Mo)-lamellae^1^.

At high solidification rates (> 25.4 mm/h) in a Bridgman apparatus, the solid/liquid interface becomes unstable and a cellular microstructure forms^2,12^. This critical withdrawal rate depends on the content of Mo and on potential impurities, so different thresholds have been reported^1,13^. Furthermore, fast solidification can lead to discontinuous (Cr,Mo) lamellae. Raj et al. found continuous (Cr,Mo) lamellae in a slowly solidified NiAl-33Cr-1Mo alloy, whereas interrupted lamellae are found if the solidification rate exceeds 127 mm/h^13^. Based on^13–15^, for fast solidification techniques, a cellular microstructure is expected, which demonstrated to have negative impact on the strength and fracture toughness. However, phases become smaller and more interfaces are present, leading to a positive impact on hardness, high temperature strength and fracture toughness by crack hindering mechanisms^9,13^. However, common casting techniques only provide limited temperature gradients and cooling rates (10^3^–10^5^ K/m at 10^–6^–10^–3^ m/s^12,13^), and as a result, lamellar spacings smaller than 0.39 µm have not been reported for a bulk material^16^. Only Shechtman et al. have melt spun 25 µm thin ribbons of NiAl-34Cr, containing eutectic microstructure with very fine spacings of 12 nm^17^. In the present work, electron beam remelting was used to produce a nanostructured alloy, simulating an additive manufacturing process and assessing its feasibility.

Electron beam melting (EBM) is an additive manufacturing process that is based on the selective consolidation of metal powder layers^18^. First, a recoater system is used to apply a powder layer of defined thickness. Subsequently, the powder is pre-heated by deflecting the defocused electron beam with high frequency across the build area. The pre-heating is necessary to induce slight sintering of the powder-bed, which increases its electrical conductivity and mechanical stability. These properties are required for the third step where the focused electron beam is used to selectively melt one cross-section of the desired component. The geometries of the cross-sections are obtained in advance by horizontal slicing of a digital model of the desired component. After the melting step, the build platform is lowered by the layer thickness and a new powder layer is applied. The cycle is repeated until all cross-sections are processed and the entire component is generated. This layer-wise approach enables an economic, tool-free production of complex parts in small batches, which is an important aspect for the application of EBM in aerospace and medical industry. Additionally, EBM has shown the ability to process demanding materials, e.g. non-weldable nickel-based superalloys where a significant downscaling of microstructural features was reported^19^. This is due to the small volume of the melt pool which enables a very high cooling rate during solidification. By adjusting the beam parameters (beam power, deflection speed, etc.), the energy input and hence solidification conditions (temperature gradient, cooling rate, etc.) can be controlled to realize selective tailoring of the microstructure^20^. The very high cooling rates and temperature gradients (10^8^–10^9^ K/m at 10^4^–10^5^ m/s^18^) exceed by far the values in common casting techniques resulting in an extremely fine microstructure. Therefore, EBM is considered a promising technique for processing of NiAl-(Cr,Mo) alloys and first investigations into its feasibility are presented in the current study.

## Materials and methods

A NiAl-28Cr-6Mo (at.-%) in-situ composite was cast using the Bridgman process with a growth rate of 1 mm/min to create bars of 11.9 mm diameter^21^.

A cross-sectional slice of the ingot with a size of 11.9 × 50 × 5 mm^3^ was prepared and placed onto a standard EBM steel base-plate in approximate central position of the build area. The exact position of the specimen with respect to the electron beam was determined by using in-situ electron optical imaging^22^ and used to calculate the sample coordinates for subsequent remelting. Remelting was performed under high vacuum conditions at room temperature in an in-house developed EBM system with an electron beam gun by pro-beam GmbH & Co. KGaA (Gilching, Germany) and by using a beam current of 7 mA and an acceleration voltage of 60 kV. Four quadratic samples with each a side length of 10 mm and a spacing of 2 mm were molten in adjacent position onto the specimen. While the scan velocity was varied from 1,000, 500, 250 to 125 mm/s, the scan strategy was always a standard EBM hatch pattern with a line spacing of 100 µm and a rotation of 180° between adjacent lines. The samples were molten in direct succession without temporal interruption, starting with the highest scan velocity (i.e. lowest energy input) to minimize the effect of specimen heat-up on the solidification conditions. The microstructures were analysed using a Zeiss Crossbeam 540 FIB/SEM (Zeiss GmbH, Oberkochen, Germany). Since the phases present in the material exhibit a significant difference in atomic number, imaging back-scattered electrons (BSE) was used. Chemical composition was analysed via energy dispersive X-ray spectroscopy (EDS) with an INCA PentaFET-X3 from Oxford Instruments.

Atom probe specimen were produced by in-situ lift-out using a Zeiss Crossbeam 540 FIB/SEM as described in^23,24^. The APT experiments were carried out in a CAMECA LEAP 4000X HR (CAMECA Inc, Madison, Wisconsin) using pulsed voltage and laser mode to trigger field evaporation. Voltage pulsing was used for more accurate determination of the chemical composition while laser pulsed experiments, yielding larger datasets, were used to show the three dimensional nanostructure for all samples. The voltage was regulated to detect an ion on average in 1% of the pulses, with the pulse voltage set to 20% of the direct current standing voltage. For the laser pulsed experiments, a UV-laser with 355 nm wavelength was used, at a pulse energy of 75 pJ. The experiment (base) temperature was 49 K and a pulse repetition rate of 200 kHz for both voltage pulsed and laser pulsed experiments. The data processing was done using the commercial software IVAS 3.6.8 from CAMECA, implementing a reconstruction algorithm published in^25,26^.

The microhardness was determined with a NanoXP Nanoindenter from Keysight (Keysight Technologies Inc., Santa Rosa, CA, USA) using a Berkovich indenter tip geometry. The maximum indentation depth was 500 nm and the hardness was averaged over the depth range from 400 to 500 nm.

## Results

### Microstructure

**Figure 1:**
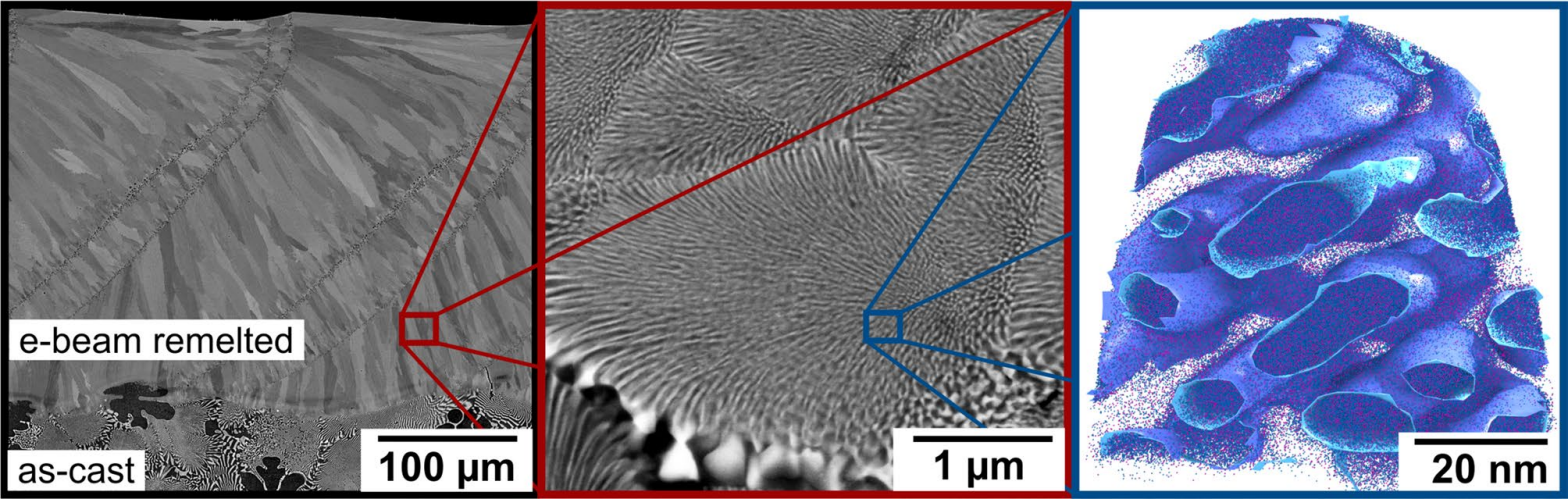
BSE micrographs showing the cross section of the remelted layer processed with 250 mm/s scan speed on top of the as-cast material. A cellular microstructure containing NiAl- (dark) and (Cr,Mo)-phases (light) forms. Atom probe tomography visualizes the (Cr,Mo)-phase by the 40 at.-% isoconcentration surface. Displayed are Cr- (blue) and Mo-atoms (purple).

The e-beam remelted layer exhibits similar microstructural features for all tested specimen. Figure 1 shows BSE cross sections of the sample fabricated with a scan speed of 250 mm/s. The nanostructured remelted layer was formed on top of the coarse as-cast microstructure. In the e-beam remelted layer, all samples show a cellular microstructure, where solidification along the temperature gradient leads to the formation of a columnar texture. The cells start growing from the solid liquid interface during remelting, visible at those dark lines perpendicular to the directed cellular texture in Fig. 1. These lines indicate the u-shaped boundaries of the melt pool during electron beam remelting and are visible due to the formation of coarser spherical NiAl and (Cr,Mo) precipitates. The Nucleation rate is very high at the boundary of the melt pool and subsequent heat flux leads to coarsened phases. Their linear arrangement within a distance of 100 µm parallel to the scanning direction forms according to the preset line distance of the e-beam. The microstructure in Fig. 1 exhibits coarser phases at the cell boundaries and fine phases inside the cells that are curved towards the centre. (Cr,Mo)-phase forms a cross-linked network inside a NiAl-matrix. The rate of solidification is too high to create a stable solid liquid interface in order to get planar lamellae. The coarse as-cast material with phase distances of 2.039 ± 1.213 µm and primarily solidified NiAl-dendrites differs from the fine-structured remelted layer.

**Figure 2:**
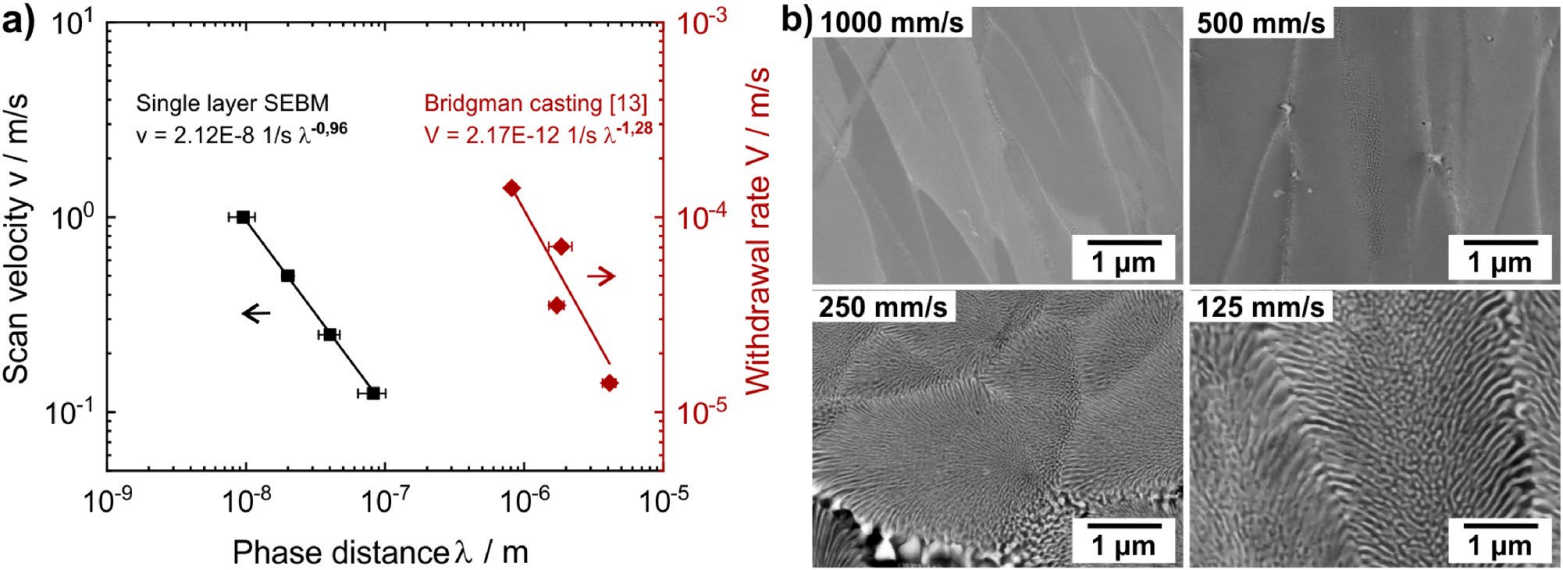
(**a**) Phase distance of NiAl- and (Cr,Mo)-phases as a function of the scan rate in e-beam remelting and the withdrawal rate of a Bridgman apparatus respectively. Data for Bridgman casting were published in^13^. (**b**) BSE micrographs of all samples processed via e-beam remelting.

Figure 2 shows the decrease in cell size and phase distance with increasing scan velocity. For high scan rates at constant beam power, the energy input into the material is lower and the melt pool is smaller. As a result the layer thickness decreases, the solidification rate increases and finer phases form. For 1,000 mm/s the phase distance is 9 ± 2 nm, whereas for 125 mm/s coarser phase distance of 82 ± 19 nm are measured. In comparison to the directional solidification in a Bridgman process from Raj et al.^13^, where the phase distances are in a range from 0.8 to 4.1 µm a much finer microstructure is obtained. A linear relationship in the double logarithmic diagram is found for both processes, which is in agreement with the Jackson-Hunt Model, that describes the lamellar and rod-like growth in eutectic alloys^27^.

**Figure 3:**
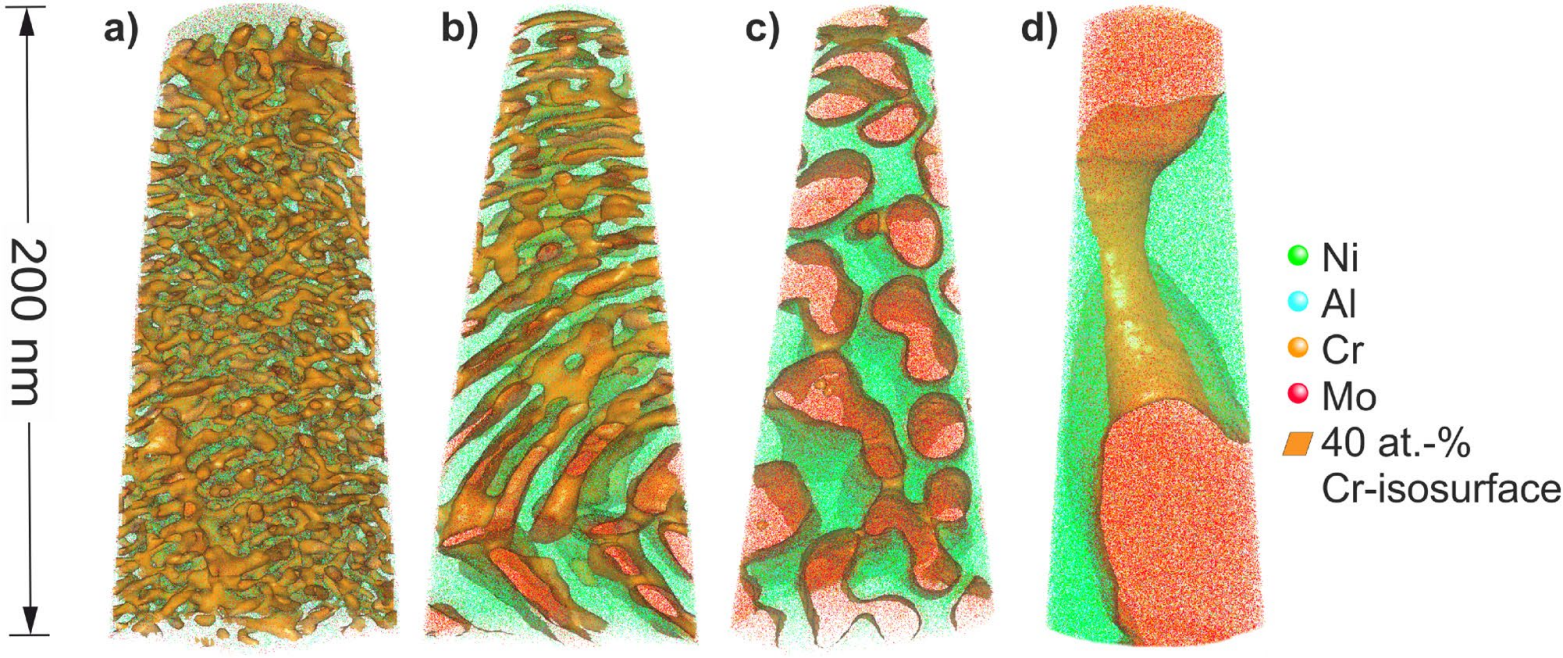
Atom probe tomographies of the nanostructured e-beam remelted layer processed by different scan speeds: (**a**) 1,000 mm/s, (**b**) 500 mm/s, (**c**) 250 mm/s, (**d**) 125 mm/s.

Previous investigations on NiAl-28Cr-6Mo reveal lamellar microstructures for all withdrawal rates^8,9,28^. In this study, the two-phase microstructure does not seem to be continuously lamellar. Figure 3 shows a coral-like arrangement of the cross-linked (Cr,Mo)-phase inside the NiAl-matrix. APT measurements support the trend observed in Fig. 2 of refining microstructure with increasing scan velocity. In Fig. 3a,b coarser cell boundaries as well as the shift in phase orientation at cell boundaries can be observed in the lower part of the tomographies.

### Chemical phase analysis

EDS-measurements reveal a homogeneous composition throughout the remelted layer. There are no concentration gradients towards the surface or the as-cast material. The average composition of the remelted layer with a scan speed of 250 mm/s is near eutectic (Table 1). For other samples a minor trend of Cr-evaporation with decreasing scan speed can be observed. The lack of Al originates from evaporation during the casting process, since both as-cast and e-beam remelted layer exhibit similar composition. Despite of non-eutectic composition, the remelted layer exhibits a eutectic microstructure. The fast solidification enables the creation of a eutectic-like microstructure in a hypereutectic alloy, as it is described by Shang et al.^12^.

**Table 1:** Phase specific composition of the NiAl- and Cr-phase, determined by voltage pulsed atom probe tomography experiments.

Phase	Scan speed (mm/s)	Ni (at.-%)	Al (at.-%)	Cr (at.-%)	Mo (at.-%)	Si (at.-%)	C (at.-%)
NiAl	1,000	48.14	41.89	6.90	2.87	0.08	0.03
	500	50.48	44.00	3.58	1.62	0.05	0.03
	250	50.25	45.90	2.35	1.35	0.06	0.01
	125	51.17	46.58	1.36	0.67	0.03	0.02
(Cr,Mo)	1,000	6.25	5.86	66.28	20.85	0.34	0.08
	500	4.63	5.14	68.47	20.88	0.26	0.06
	250	2.27	2.33	71.66	23.04	0.29	0.02
	125	1.84	1.58	71.60	21.91	0.26	0.09

The phase specific chemical composition is determined using the partitioning ratio *k*_*i*_ (Eq. (1)) of the NiAl- and (Cr,Mo)-phases.

1 $$k_{i}^{{NiAl/Cr\left( {Mo} \right)}} = \frac{{c_{i}^{NiAl} }}{{c_{i}^{{\left( {Cr,Mo} \right)}} }}$$

$c_{i}^{NiAl}$, $c_{i}^{{Cr\left( {Mo} \right)}}$: concentrations of the element $i$ in the NiAl- and (Cr,Mo)-phase.

**Figure 4:**
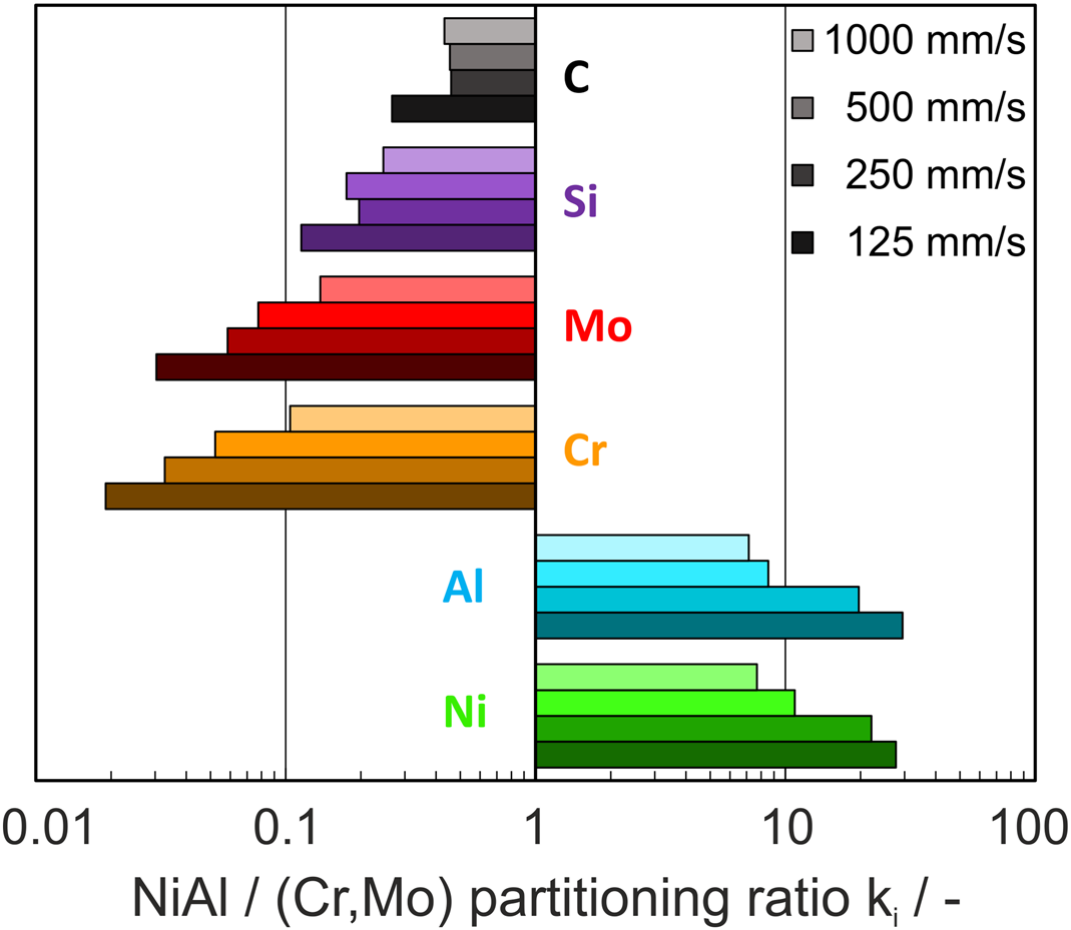
Partitioning ratio of the main elements Ni, Al, Cr and Mo, as well as impurities Si and C in NiAl- and (Cr,Mo)-phases.

The partitioning ratio of Ni and Al as well as Cr and Mo is similar at different scan rates (Fig. 4). The solubility of foreign atoms increases with faster scan velocities. The solubility of Cr in NiAl at the highest scan speed is five times the solubility at the slowest scan speed. This is consistent with the trend observed by Sheng et al.^16^. However, the amount of foreign atoms is quiet low, compared to those in as-cast NiAl-(Cr,Mo) observed in^29^. The NiAl-phase exhibits a lack of Al and a light surplus of Ni in comparison with the stoichiometric ratio. The (Cr,Mo)-phase shows a lack of Cr due to evaporation and therefore a surplus of Mo. Minor contaminations of C and Si were introduced during the casting process without purpose. They strongly segregate in the (Cr,Mo)-phase. Especially for C, a preferred accretion at the phase and cell boundaries can be observed.

### Microhardness

**Figure 5:**
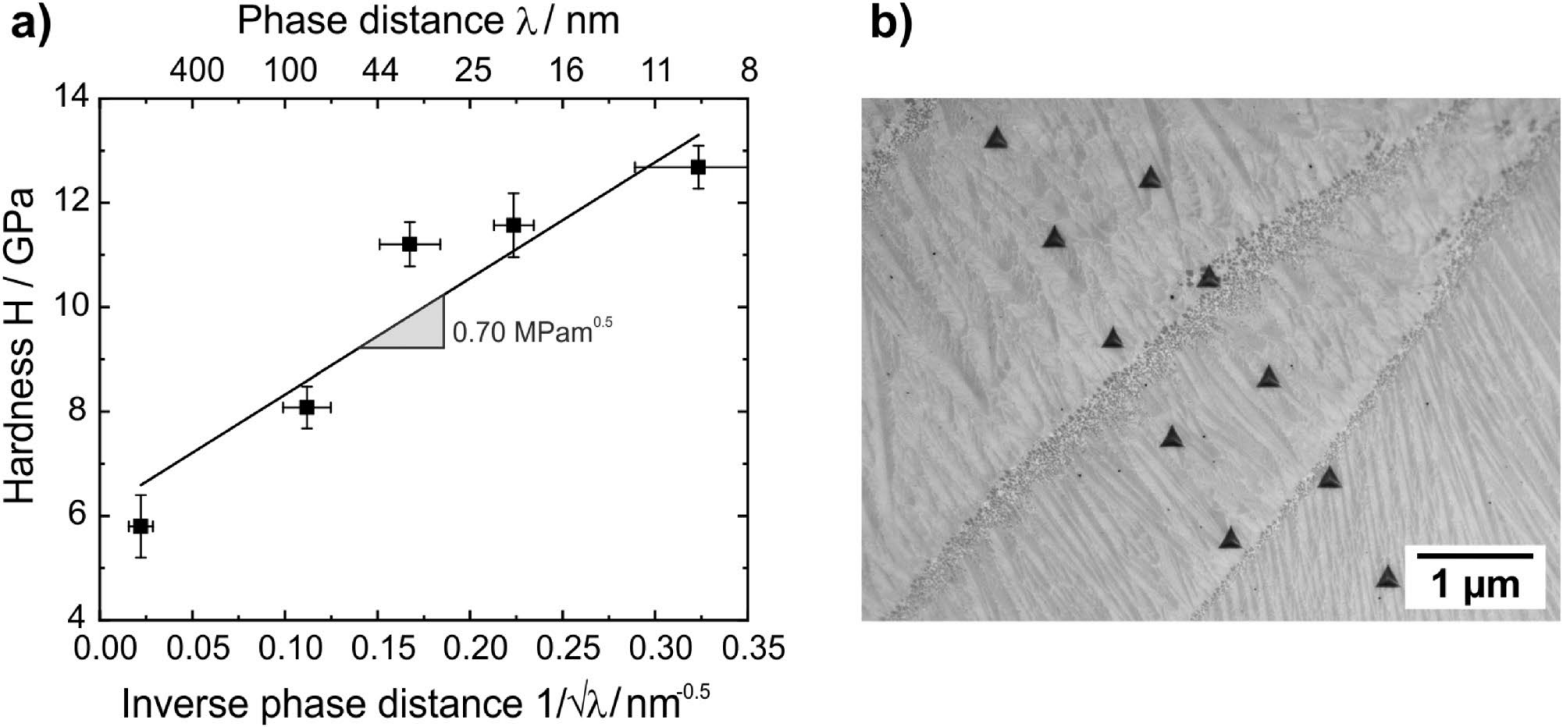
(**a**) Increasing hardness with decreasing phase distance. (**b**) Series of Nanoindents across the remelted layer.

The hardness of remelted layer approaches 7.7–12.7 GPa, which is significantly harder than the as-cast material (5.2 GPa for indents in the NiAl-phase and 6.7 GPa for indents in the (Cr,Mo)-phase). The highest hardness is found for the finest microstructure. Figure 5 displays the hardness as a function of the inverse square root of the phase distance. A linear relationship can be found in this plot, similar to grain boundary strengthening as described by Hall–Petch^30^. According to (Eq. (2)), the Hall–Petch constant $k$ can be determined to be 0.70 MPam^1/2^, the hardness $H_{0}$ equals 6,116 MPa. $\lambda$ represents the phase distance.

2 $$H = H_{0} + k*\frac{1}{\sqrt \lambda }$$

Another possible impact increasing the hardness difference of the as-cast material and the electron beam remelted layers is the presence of high residual stresses. High cooling rates in electron beam melting can create thermally induced stresses, especially for high melting intermetallic materials. However, the influence of residual stresses on hardness measurements is not so high and typically below 10%^31^ and this probably plays only a minor role here.

### Crack propagation

**Figure 6:**
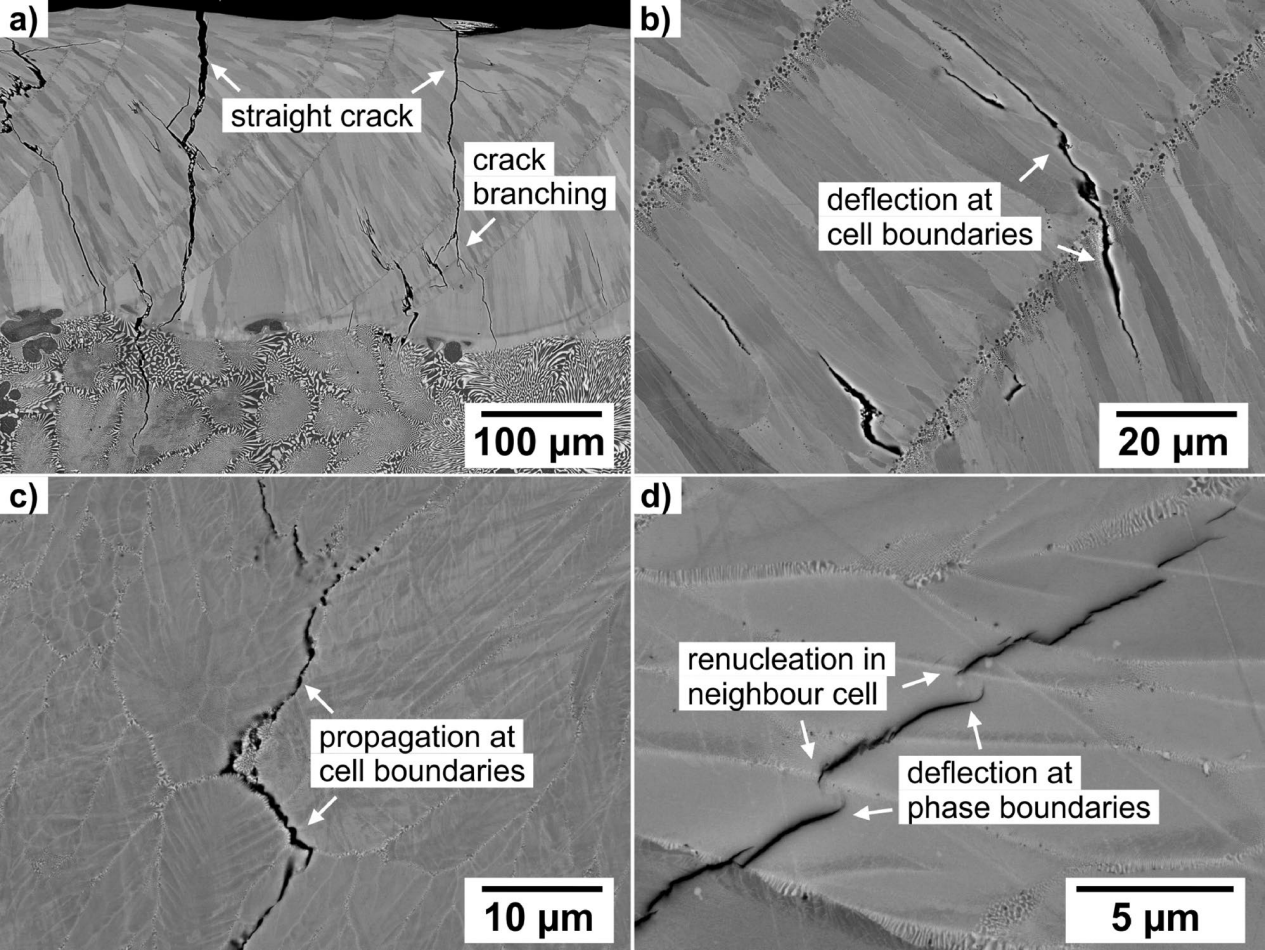
Crack-hindering mechanisms in the remelted layer at different scales: (**a**) crack branching, (**b**, **c**) crack deflection along cell boundaries, (**d**) crack deflection along phase boundaries.

The as-cast material was not preheated in advance of electron beam remelting, so high thermal stresses caused by thermal shock, fast solidification and volume shrinkage were introduced. At slow scan velocities the energy input is high, so the whole sample will heat up and thermal stresses and crack formation decreases. Figure 6a exhibits large cracks that cut the remelted layer perpendicular. They stop in the coarse as-cast microstructure where the crack is deflected along the phase boundaries or energy is dissipated by plastic deformation of the (Cr,Mo)-phase. These crack-hindering mechanisms are already described elsewhere^8,9,13,14,32^. Figure 6b,c also show crack deflection in the e-beam remelted layer along the columnar texture parallel to the solidification direction. Cracks branch and are forced in energetically unfavourable directions. At high magnifications, crack deflection along nanoscaled phase boundaries inside the cells can be observed. The cracks follow the feather-like phases towards cell centre. The change in phase orientation leads to crack stopping and crack renucleation in the neighbouring cell with a more favourable phase orientation.

## Discussion

The nanostructured phase alignment of NiAl- and (Cr,Mo)-phases is achieved by the very fast solidification rate. The eutectic solidification as well as the low miscibility of both phases enables phase distances below 10 nm. It should be noted that phase distances were determined from BSE images, leading to systematic overestimation of the values. The relationship between the phase distance and the solidification rate according to Fig. 2 can be described by (Eq. (3)):

3 $$v = A {\uplambda }^{B} { }$$

with λ representing the phase distance in m. For electron beam remelting, the velocity $v$ is the scan rate of the e-beam in m/s, whereas for directional solidification $v$ represents the withdrawal rate in m/s. From Fig. 2 the parameters A and B have been determined to be $2.12 \times 10^{ - 8} 1/{\text{s}}$ and − $0.96$ for e-beam remelting. For Bridgman casting according to^13^, the parameters are $2.17 \times 10^{ - 12} 1/{\text{s}}$ and − $1.28$. Shang et al. found that the Jackson-Hunt model is applicable not only for planar eutectics, but also for cellular eutectic alloys like in this study^12^. An accurate measurement of the temperature or the cooling rate in the harsh environment of electron beam melting is currently not possible. To overcome this gap, material specific numerical approaches can be used to simulate the process and deduce information about solidification conditions^33^.

The preferred lamellar microstructure could not be formed like it is present in as-cast alloys due to fast solidification^29^. Nucleation rate is increased, diffusion is suppressed, leading to prevention of lamellar growth. The (Cr,Mo)-phases in Figs. 2 and 3 are in fact cross-linked rods with feather-like arrangement inside the cells. The cross-linkage is anisotropic and cross sections of (Cr,Mo)-rods are not spherical. This indicates a tendency for the aimed lamellar arrangement. The phases are small at the cell center and coarsen towards the cell boundary. The solidification slows down towards the cell boundary, due to the recalescence released at the solid–liquid interface as described in^29^. Another influence on phase morphology is given by the melting range. Eutectic alloys, such as 33.2Ni–33.2Al–33.6Cr, solidify with a sudden liquid–solid transition^11^. The lack of Cr and Al as well as the presence of Si and C cause deviation off the eutectic. Local concentration gradients will provide undercooling at the solid–liquid interface, leading to instability and non-planarity of the interface. In this case, a continuous lamellar morphology is not possible. On top of that, the substitution of 6 at.-% Cr with Mo affects the eutectic point^27,34^.

Figure 1 shows columnar cells growing along the temperature gradient. This directed microstructure is often observed in powder bed electron beam melting processes. As reported in many researches^19,35,36^, this texture leads to anisotropic behaviour of mechanical properties. In the case of this work, columnar cells consists of smaller, more equiaxed cells (see Fig. 2), leading to frequent interruption of the columnar cells. With the hardness measurements performed in this work, no influence of the orientation on the hardness has been detected. In general, influences of the orientation on nanoindentation measurements are often strongly visible on the different pile-up pattern, however not so strong on the absolute hardness numbers, as has been reported for example on lamellar TiAl alloys^37,38^. Other mechanical properties like fracture toughness will show stronger anisotropy, as can be seen in experiments on NiAl single crystals^39^.

For as-cast NiAl-28Cr-6Mo more than 20 MPam^1/2^ was reported in^8,9^. In this work, fracture toughness was not determined, but similar crack deflection and crack hindering mechanisms were found. Coarse phases at the cell boundaries represent a preferred crack path. In case of a columnar textured material processed by electron beam remelting, the cracks are deflected alongside those columnar cells. Depending on the orientation of the columnar cell structure, anisotropic fracture behaviour is expected. Higher fracture toughness will appear for crack propagation perpendicular to the columnar texture. The fine feather-like phases exhibit crack deflecting potential to prevent crack growth perpendicular to the phases. In addition, similar crystal structures and lattice parameters of the NiAl- and the (Cr,Mo)-phase lead to a strong interfacial bonding. Figure 6d indicates, that cracks will rather be deflected along the NiAl–(Cr,Mo) interface than propagate perpendicular to the phases and interphases. Plastic deformation of the more ductile (Cr,Mo)-phase was not observed. Since cracks, which are formed in the remelted layer are stopped in the base material, fracture toughness of remelted material is expected to be smaller, but toughening mechanisms are still present.

For common powder bed selective electron beam melting, the sample usually is preheated to control residual stresses and annihilate crack formation in the material during processing^18^.

## Summary and conclusion

This study is a first promising approach towards additive manufacturing of nanoscaled NiAl-(Cr,Mo) in-situ composites. The effects of fast solidification on microstructure and mechanical properties of the in-situ composite NiAl-28Cr-6Mo were analysed and the following results and conclusions were found:

- E-beam remelted composites consists of cellular microstructure with a feather-like arrangement of (Cr,Mo)-rods in NiAl-matrix. The preferred lamellar growth is supressed by fast solidification.
- A non-eutectic alloy can solidify with a eutectic-like microstructure for high solidification rates.
- The nanostructured material with phase distances less than 10 nm exhibits superior hardness up to twice the hardness of the as-cast material, that can be described by the Hall–Petch relation.
- Crack hindering mechanisms on different microstructural scales are present. The preferred crack path is the coarse cell boundary and the coral-like (Cr,Mo) network shows crack deflection.
